# Review of the evidence regarding the use of antenatal multiple micronutrient supplementation in low‐ and middle‐income countries

**DOI:** 10.1111/nyas.14121

**Published:** 2019-05-27

**Authors:** Megan W. Bourassa, Saskia J.M. Osendarp, Seth Adu‐Afarwuah, Saima Ahmed, Clayton Ajello, Gilles Bergeron, Robert Black, Parul Christian, Simon Cousens, Saskia de Pee, Kathryn G. Dewey, Shams El Arifeen, Reina Engle‐Stone, Alison Fleet, Alison D. Gernand, John Hoddinott, Rolf Klemm, Klaus Kraemer, Roland Kupka, Erin McLean, Sophie E. Moore, Lynnette M. Neufeld, Lars‐Åke Persson, Kathleen M. Rasmussen, Anuraj H. Shankar, Emily Smith, Christopher R. Sudfeld, Emorn Udomkesmalee, Stephen A. Vosti

**Affiliations:** ^1^ The New York Academy of Sciences New York New York; ^2^ Osendarp Nutrition Berkel & Rodenrijs the Netherlands; ^3^ Division of Human Nutrition and Health Wageningen University Wageningen the Netherlands; ^4^ Department of Nutrition and Food Science University of Ghana Legon Accra Ghana; ^5^ The Vitamin Angels Alliance, Inc. Santa Barbara California; ^6^ Johns Hopkins Bloomberg School of Public Health Baltimore Maryland; ^7^ Bill & Melinda Gates Foundation Seattle Washington; ^8^ London School of Hygiene and Tropical Medicine London United Kingdom; ^9^ UN World Food Programme Rome Italy; ^10^ Friedman School of Nutrition Science and Policy Tufts University Boston Massachusetts; ^11^ Department of Nutrition University of California Davis Davis California; ^12^ International Centre for Diarrhoeal Disease Research Dhaka Bangladesh; ^13^ UNICEF Copenhagen Denmark; ^14^ The Pennsylvania State University, State College Pennsylvania; ^15^ Division of Nutritional Sciences Cornell University Ithaca New York; ^16^ Helen Keller International Baltimore Maryland; ^17^ Sight and Life Foundation Basel Switzerland; ^18^ UNICEF New York New York; ^19^ Department of Women and Children's Health King's College London London United Kingdom; ^20^ Global Alliance for Improved Nutrition GAIN Geneva Switzerland; ^21^ London School of Hygiene and Tropical Medicine Addis Ababa Ethiopia; ^22^ Department of Global Health and Population Harvard T.H. Chan School of Public Health Boston Massachusetts; ^23^ Summit Institute of Development Mataram Indonesia; ^24^ Institute of Nutrition Mahidol University Nakhon Pathom Thailand

**Keywords:** LMICs, micronutrient, supplements, pregnancy

## Abstract

Inadequate micronutrient intakes are relatively common in low‐ and middle‐income countries (LMICs), especially among pregnant women, who have increased micronutrient requirements. This can lead to an increase in adverse pregnancy and birth outcomes. This review presents the conclusions of a task force that set out to assess the prevalence of inadequate micronutrient intakes and adverse birth outcomes in LMICs; the data from trials comparing multiple micronutrient supplements (MMS) that contain iron and folic acid (IFA) with IFA supplements alone; the risks of reaching the upper intake levels with MMS; and the cost‐effectiveness of MMS compared with IFA. Recent meta‐analyses demonstrate that MMS can reduce the risks of preterm birth, low birth weight, and small for gestational age in comparison with IFA alone. An individual‐participant data meta‐analysis also revealed even greater benefits for anemic and underweight women and female infants. Importantly, there was no increased risk of harm for the pregnant women or their infants with MMS. These data suggest that countries with inadequate micronutrient intakes should consider supplementing pregnant women with MMS as a cost‐effective method to reduce the risk of adverse birth outcomes.

## Graphical abstract

This review presents the conclusions of a task force that set out to provide guidance to countries considering using multiple micronutrient supplements in pregnant women by assessing the available evidence.

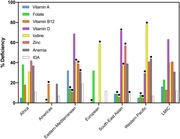

## Introduction

Adequate intakes of essential vitamins and minerals (micronutrients) are required during pregnancy for maternal health and fetal development. Insufficient nutrient intakes before and during pregnancy, combined with increased metabolic demands during pregnancy, can result in individuals suffering from one or more micronutrient deficiencies, especially in low‐ and middle‐income countries (LMICs). Supplementation with multiple micronutrient supplements (MMS) that include iron and folic acid (IFA) during pregnancy is practiced in some countries, including the United States and South Africa, to increase micronutrient intakes and improve pregnancy outcomes.^1^, ^2^ However, IFA supplementation is currently the recommended standard of care for pregnant women in many countries.

Two recent reviews, a Cochrane systematic review and meta‐analysis and an individual‐participant data (IPD) meta‐analysis, have assessed trials that compared the use of MMS with IFA in pregnant women and were predominantly conducted in LMICs.^3^, ^4^ Both reviews reported improved pregnancy and birth outcomes among women receiving MMS, including a lower incidence of low birth weight (LBW) and small for gestational age (SGA) births as compared with women receiving IFA supplementation. An earlier (2015) version of the *Cochrane Review* informed the 2016 World Health Organization (WHO) Guidelines for Antenatal Care (ANC), while the IPD meta‐analysis was published after the guideline committee meetings.

The current WHO ANC Guidelines do not recommend that MMS replace IFA as routine standard of care because of “… some evidence of risk, and some important gaps in the evidence.”^5^ However, the guidelines further comment “that policymakers in populations with a high prevalence of nutritional deficiencies might consider the benefits of multiple micronutrient supplements on maternal health to outweigh the disadvantages [such as cost], and may choose to give multiple micronutrient supplements that include iron and folic acid.” Yet, no further guidance was provided regarding the contexts where MMS may be warranted.

Two exceptions to this guideline are in emergency settings and for pregnant women with active tuberculosis (TB), where the risk of micronutrient deficiencies is especially high in vulnerable groups, including pregnant and lactating women. To address these risks in emergency settings, the WHO, UNICEF, and WFP issued a statement in 2007 recommending MMS for pregnant and lactating women.^6^ The recommendation states that MMS should be given in addition to IFA in settings where IFA supplementation exists, even in the presence of fortified rations, and should continue for the duration of the emergency. In the case of active TB, WHO guidelines from 2013 mandate MMS for pregnant and lactating women for nutritional care and support for patients.^7^

This review is based on the conclusions of a task force created to develop guidance for countries as they consider implementation of MMS as a standard of care for pregnant women and to assess the benefits and possible risks of antenatal MMS use in LMICs. The task force, comprising 33 members from a variety of organizations, including nongovernmental organizations, nonprofit organizations, and academia, met in November 2017 and April 2018 (see Appendix 1 for a full list of members of the task force; online only). This work was commissioned by the Bill & Melinda Gates Foundation and organized by the New York Academy of Sciences. As a direct outcome of the task force, the objectives of the current review are to (1) review the evidence on the burden of micronutrient deficiencies in women of reproductive age (WRA) and associated adverse pregnancy outcomes; (2) update the evidence base on the effects of MMS on pregnancy outcomes; (3) assess the costs and cost‐effectiveness of shifting from IFA to MMS during pregnancy; and (4) provide indicators based on the updated evidence base that might be considered by governments deciding whether IFA or MMS is the most appropriate supplement for their contexts.

## The role of micronutrients in pregnancy

As discussed in a recent detailed review, the importance of several individual micronutrients during critical periods of human development is reflected in the evidence from human trials and observational data.^8^ Much of our understanding of the mechanisms by which micronutrients affect critical early pregnancy events is derived from animal models. Micronutrients are known to affect conceptus development from early gestation onward, including implantation and vascularization of the placenta, offspring morphogenesis, organogenesis, and neurological development.^8^

Specifically, zinc, folate, niacin, riboflavin, and vitamins B_6_ and B_12_ are considered particularly important in early gestation as these micronutrients are involved in one‐carbon metabolism, which is essential for cell proliferation, growth, and protein synthesis in the earliest stages of gestation.^8^, ^9^ These micronutrients are also known to play a role in the rapid demethylation of maternal and paternal genomes immediately following conception, as well as the sustained remethylation of the fetal genome during gestation. Zinc and vitamin D also contribute to placental development and function. Throughout gestation iron, folate, zinc, niacin, and vitamins B_6_ and A support the organogenesis and development of the fetal central nervous system, while iodine is important in early brain development.^8^, ^9^ A recent study demonstrated that mitochondrial efficiency was greater for women receiving MMS compared with IFA.^65^ Thus, micronutrient deficiencies can result in a wide variety of birth defects and adverse pregnancy outcomes, including LBW, which is caused by either intrauterine growth restriction (IUGR) or preterm birth.

## Micronutrient deficiencies during pregnancy

Monotonous, nutrient‐poor diets are common among women in LMICs, and the increased nutrient needs of pregnancy further raise the likelihood that pregnant women in settings with low dietary diversity or food insecurity will experience inadequate intakes of micronutrients as well as energy, essential amino acids, and fatty acids. While the focus of this review is on micronutrient nutrition during pregnancy, it is important to acknowledge the coexistence of protein and energy deficiencies along with micronutrient deficiencies, which may further exacerbate the adverse effects on pregnancy outcomes. In addition to IFA supplements, the WHO ANC Guidelines therefore make a context‐specific recommendation for balanced energy and protein dietary supplementation for pregnant women in undernourished populations to reduce the risk of stillbirths and SGA neonates.^10^, ^11^

Micronutrient deficiencies are prevalent among pregnant and lactating women and WRA in LMICs, although data are limited. A systematic review of studies reporting on selected vitamin and mineral intakes among pregnant women in LMICs concluded that inadequate nutrient intakes from poor diets were common, with mean/median folate and iron intakes most frequently found to be below the estimated average requirements, followed by calcium and zinc.^12^ Estimates reported in 2013 by Black *et al*. suggest that 19.2% of pregnant women in LMICs had iron deficiency anemia (IDA) on the basis of 2011 data, 15.3% were vitamin A deficient (data from 1995 to 2005), and 17.3% were at risk of zinc deficiency (based on inadequate zinc intakes from 2005).^13^

**Figure 1:**
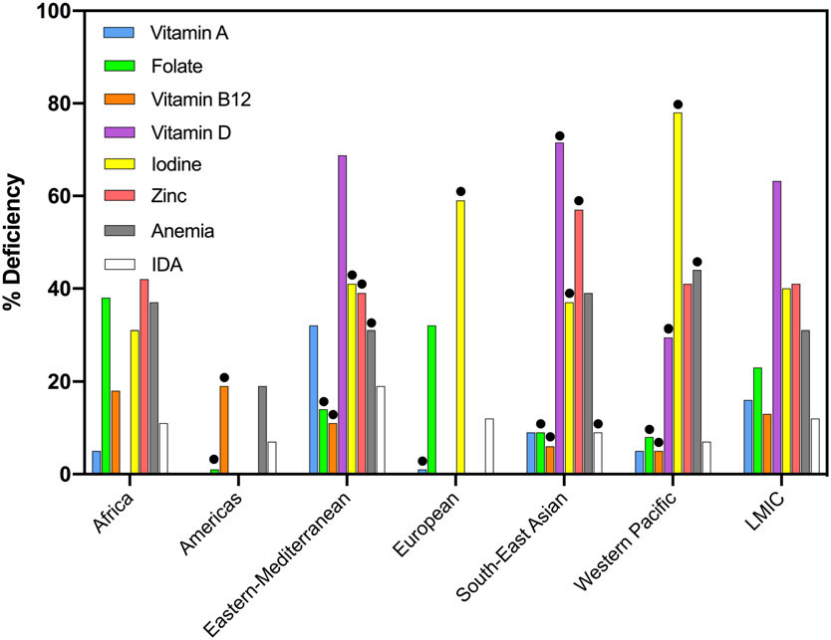
Regional estimates of micronutrient deficiencies and anemia as reported in women of reproductive age. Black circles are not representative (<3 countries). Data calculated from 52 national and regional surveys, published between 2013 and July 2017.^66^ Missing bars means no data were found for that micronutrient in the specific region.

**Figure 2:**
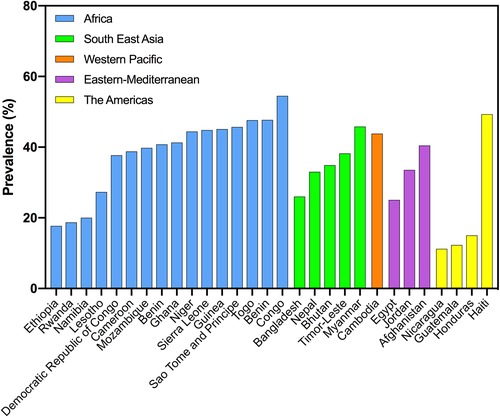
Prevalence of anemia (Hb < 120 g/L) by country among women of reproductive age in LMICs. Data calculated from 52 national and regional surveys, published between 2013 and July 2017.^66^

An update of the Black *et al*. review,^13^ including data from WRA in addition to data from pregnant women, was performed for this task force by searching for surveys published from 2013 until July 2017 in PubMed. The search strategy and number of articles identified are described in Appendix 2 (online only). The percentage of deficiencies was used as reported in the survey, even though cutoffs used to define deficiencies varied among the studies and surveys. Cutoffs used for each survey are described in Appendix 3 (online only) for WRA. In general, deficiencies of vitamin D, iodine, and zinc were widespread in WRA (Fig. 1). On the basis of the individual reported country prevalences, weighted average regional (using WHO regions) prevalences were calculated, if there were at least three country estimates available for the particular micronutrient deficiency. On average, 63.2% of WRA were vitamin D deficient, 41.4% were zinc deficient, 31.2% were anemic, and 22.7% were folate deficient. Iron deficiency could not be estimated, but IDA was reported in some studies and was often less than half of all anemia (Fig. 1). The prevalence of vitamin A deficiency in WRA was 15.9% for all LMICs but varied substantially between countries. For instance, in Pakistan, 43.2% of all WRA were reported to be vitamin A deficient,^14^ whereas the prevalence was only 1.6% in Vietnam.^15^ Similarly, there was a high variability in anemia prevalence among countries. In Africa, estimates of anemia prevalence in WRA ranged from 17.7% in Ethiopia^16^ to as high as 54.5% in Congo^17^ (Fig. 2). It should be noted that although many of the surveys are more than 10 years old and updated data on micronutrient deficiencies among women and children are needed, available evidence indicates that multiple micronutrient deficiency is a public health problem in most LMICs.

**Figure 3:**
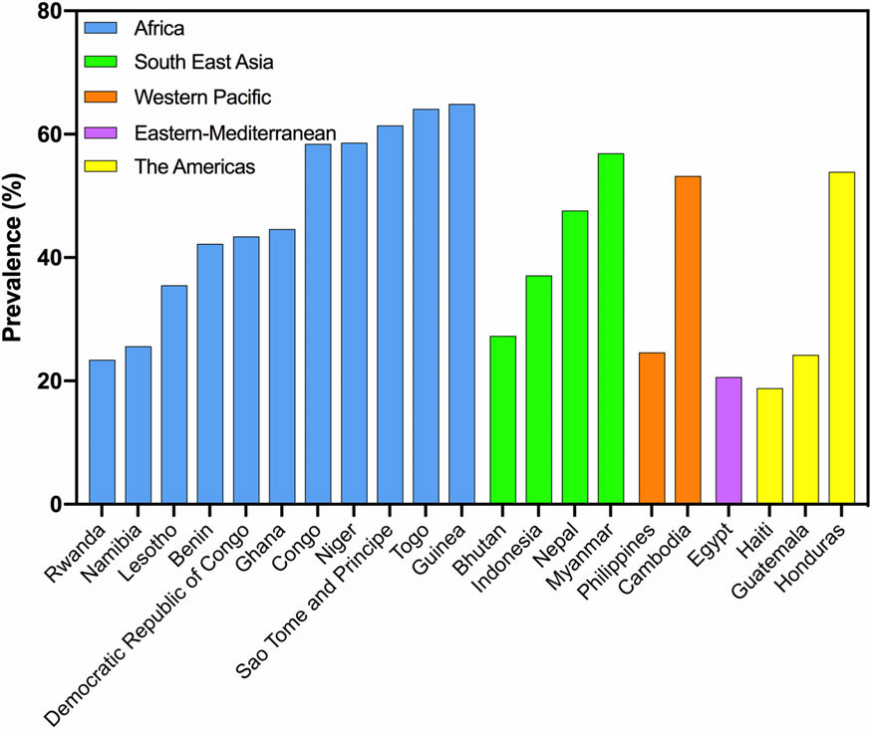
Prevalence of anemia (Hb < 120 g/L) by country among pregnant women in LMICs. Data calculated from 21 national and regional surveys and studies, from 2013 to July 2017.^66^

No regional estimates could be calculated for micronutrient deficiencies in pregnant women, with the exception of IDA and iodine, owing to a lack of data. Overall, 31.6% of pregnant women in LMICs were anemic, with particularly high prevalence in Africa (48.3%) and South‐East Asia (44.8%) (Fig. 3). Cutoffs used for each survey in pregnancy are described in Appendix 4 (online only). Assuming 50% of all anemia is caused by iron deficiency, Black *et al*. estimated regional prevalences of IDA among pregnant women of 20.3% in Africa and 19.8% in Southeast Asia. Using this same assumption to estimate IDA, our estimates in pregnant women based on the new review were 15.8% in all LMICs, with regional estimates in Africa and Southeast Asia of 24.2% and 22.4%, respectively.

The Iodine Global Network (IGN) provides the most up‐to‐date estimates for prevalences of insufficient iodine intakes and these data were used for our review. In 2017, IGN estimated that pregnant women in 40 countries had insufficient iodine intakes (median urinary iodine concentration (mUIC) <150 μg/L), particularly in South Asia, the Middle East, Europe, the United States, and Australia. In contrast, pregnant women in countries in Latin America, Southeast Asia, and Southern Africa were estimated to have adequate iodine intakes, which may partly reflect the success of salt iodization programs in these countries.^18^ It should be noted that not all data are nationally representative, and in some cases IGN relies only on subnational data to produce national estimates of iodine intake.

## Adverse pregnancy, maternal, and child outcomes

Adverse pregnancy outcomes, including LBW, preterm birth, SGA, perinatal mortality, and maternal mortality, are common in LMICs and associated with maternal micronutrient malnutrition.^8^ In addition, micronutrient deficiencies have been associated with other adverse maternal and child outcomes, including maternal depression and maternal and child cognitive impairment,^19^, ^20^, ^21^, ^22^ premature rupture of membranes, insufficient gestational weight gain, congenital anomalies, and pre‐eclampsia.^23^ Notably, stillbirth is difficult to study in LMICs because it is often not reported as an adverse outcome, but it is also associated with poor micronutrient status.

Overall, 16% of all live births in LMICs in 2014 were LBW (<2500 g) with South Asia carrying the largest burden (28%).^24^ SGA, preterm, and both preterm and SGA infants have an increased mortality risk.^25^ On the basis of secondary analyses of data from the Child Health Epidemiology Reference Group in 2012 in LMICs, an estimated 23.3 million infants, or 19.3% of all live births, were SGA; and an estimated 606,500 neonatal deaths, or 21.9% of all neonatal deaths, were attributable to SGA. SGA was defined as infants weighing <10th percentile of birth weight‐for‐gestational age and sex according to the multiethnic, INTERGROWTH‐21st birth weight standard.^26^ South Asia also had the highest prevalence of SGA (34% of all live births) (Table 1).^26^

**Table 1:** Prevalence of adverse pregnancy and birth outcomes

	Sub‐Saharan Africa	Asia	Southeast Asia	South Asia	World
Preterm births (2014) (%)^a^	12.0	10.4	N/R	N/R	10.6
SGA (2012) (%)^b^	16.5	N/R	21.6	34.2	19.3
LBW (2009–2013) (%)^c^	13.0	N/R	N/R	28	16
Maternal mortality ratio (per 100,000 live births)^d^	546	N/R	62	182	216
Stillbirths (per 1000 total births)^e^	28.7	N/R	12.2	25.5	18.4
Neonatal mortality (per 1000 live births)^f^	27.7	N/R	13.5	27.6	18.6

a Estimated mean preterm birth rate defined as all live births before 37 weeks of completed weeks of gestation, whether singleton, twin, or higher order multiples, divided by all live births in the population.^67^

b SGA, small for gestational age, defined as birth weight less than the 10th percentile for a specific completed gestational age by sex, using the INTERGROWTH‐21st standard; SGA rate defined as all term and preterm SGA, divided by all live births in the population.^26^

c Low birth weight defined as the number of live births weighing less than 2500 g divided by all live births in the population.^24^

d Maternal mortality is defined as the number of maternal deaths per 100,000 live births (maternal death = death of a women while pregnant or within 42 days of termination of pregnancy, from any cause related to or aggravated by the pregnancy or its management); 2015 estimates.^28^

e Stillbirth rate estimates are based on the late fetal death definition: 1000 g or more with an assumed equivalent of 28 weeks’ gestation or more, or 35 cm or more; 2015 estimates per 1000 total births.^29^

f Neonatal mortality defined as deaths in the first 28 days of life per 1000 live births; 2016 estimates.^30^ N/R, not reported.

A 2018 review, assessing data from National Registries, Reproductive Health Surveys and published studies, estimated that in 2014, 14.84 million babies, or 10.6% of live births, were born preterm worldwide. More than 80% of preterm babies were born in Asia (7.8 million, or 10.4% of live births) and Sub‐Saharan Africa (4.2 million, or 12.0% of live births).^27^

While conditions related to IUGR are most prevalent in South Asia, Sub‐Saharan Africa has the highest incidence of maternal mortality (546 per 100,000 live births), stillbirths (28.7 per 1000 total births), and neonatal mortality (27.7 per 1000 live births) (Table 1).^28^, ^29^, ^30^ Data on the prevalence of other adverse pregnancy outcomes, including insufficient gestational weight gain, macrosomia, pre‐eclampsia, PROM, and congenital abnormalities, are scarce and often from small, local sources, which may be prone to under‐reporting.

Poor maternal micronutrient status has also been associated with poor longer term health and development outcomes for the infant/child,^31^, ^32^ but the evidence of the impact of MMS on development is limited and exposure to other environmental risk factors later in life confounds the associations.^33^ Recent evidence, from a maternal micronutrient supplementation trial in Indonesia,^68^ suggests that children from mothers who had received multiple micronutrients during pregnancy had better cognitive development across multiple domains at 3–4 years of age, especially children of malnourished and anemic women, and improved procedural memory at 9–12 years of age. The 9‐ to 12‐year follow‐up study also found higher general intellectual ability among children of anemic mothers who received multiple micronutrients during pregnancy compared with the IFA group.^22^ Poor child development has also been indirectly associated with poor micronutrient status during pregnancy, since children born LBW are at higher risk of developmental delays later in life.^34^

## Interventions to improve micronutrient nutrition in pregnant women and to reduce adverse pregnancy and birth outcomes

To address micronutrient malnutrition in the general population, WHO and the Food and Agricultural Organization of the United Nations have identified four complementary strategies: (1) nutrition education leading to increased diversity and quality of diets; (2) food fortification and biofortification; (3) disease control measures; and (4) supplementation (currently only routine iron‐folic acid is recommended in pregnancy, along with context‐specific recommendations for protein‐energy, calcium, vitamin A, and zinc).^35^ Healthy eating is also formally recommended for pregnant women. However, it is generally a challenge to cover all of their nutrient needs with local foods because even optimized local diets may be insufficient to meet the increased nutrient needs during gestation.^8^

## Revisiting the evidence base on the effect of multiple‐micronutrient supplements in pregnancy

### Overview of the 2019 *Cochrane Review*

The 2019 *Cochrane Review* (updated from 2015) on the use of MMS during pregnancy evaluated the effects of MMS as compared with IFA on pregnancy outcomes; the 2015 *Cochrane Review* was used to inform the development of the WHO ANC Guidelines on MMS in pregnancy.^3^, ^5^ A total of 20 trials, which included a total of 141,849 women, that met the inclusion criteria and reported outcomes of interest to the review were identified. Nineteen of 20 trials were carried out in LMICs using MMS that included IFA compared with iron with or without folic acid; 14 of these trials were considered in the WHO ANC Guidelines. The Cochrane reviewers note that at the time of their literature search (February 23, 2018), there was one trial awaiting classification,^36^ as well as four ongoing trials that could not be included.^37^, ^38^, ^39^, ^40^

The analyses in the *Cochrane Review* demonstrated that overall MMS resulted in a 12% reduction in LBW (RR: 0.88; 95% CI: 0.85–0.91) and a 8% reduction in SGA births (RR: 0.92; 95% CI: 0.88–0.97), compared with IFA supplementation, with high and moderate quality evidence (based on GRADE criteria),^41^ respectively.^3^ There were no significant differences identified for other maternal or pregnancy outcomes assessed, including preterm birth, stillbirth, maternal anemia in the third trimester, miscarriage, maternal mortality, perinatal mortality, neonatal mortality, or risk of delivery by caesarean section when MMS was compared with supplementation with iron or without folic acid. A summary of these analyses is presented in Table 2, alongside the results from the IPD meta‐analysis for comparison, which is discussed in more detail below.^3^, ^4^ However, it should be noted that the IPD was based on voluntary participation of the trial's investigators and was primarily aimed at conducting subgroup analyses, while the *Cochrane Review* was focused on the overall effects of all available trials.

**Table 2:** Overall effects of MMS on birth outcomes in comparison with iron, with or without folic acid in LMICs based on the *Cochrane Review* and an IPD meta‐analysis (RR, 95% CI)

	*Cochrane Review* 3	IPD meta‐analysis^4^	
	(15 RCTs)	(17 RCTs)	
	Relative risks	Relative risks	
Outcome	Random effects	Random effects	Fixed effects
SGA (<10th percentile)	0.92 (0.88–0.97)^a^	0.94 (0.90–0.98)^b^	0.97 (0.96–0.99)^b^
LBW (<2500 g)	0.88 (0.85–0.91)	0.86 (0.81–0.92)	0.88 (0.85–0.90)
VLBW (<2000 g)	Not reported	Not reported	0.78 (0.72–0.85)
Preterm birth (<37 weeks)	0.96 (0.90–1.03)	0.93 (0.97–0.98)	0.92 (0.88–0.95)
Very preterm birth (<34 weeks)	Not reported	Not reported	0.87 (0.79–0.95)
LGA (>90th percentile Oken)	Not reported	1.04 (0.92–1.18)	1.05 (0.95–1.15)
LGA (>90th percentile INTERGROWTH)	Not reported	Not reported	1.11 (1.04–1.19)
Stillbirth	0.95 (0.86–1.04)	0.97 (0.85–1.11)	0.92 (0.86–0.99)
Neonatal mortality (≤28 days)	1.00 (0.89–1.12)	0.99 (0.89–1.09)	0.98 (0.90–1.05)
Infant mortality (≤365 days)	Not reported	0.97 (0.88–1.06)	0.97 (0.88–1.06)

a SGA defined by authors of trials.

b SGA defined by the INTERGROWTH‐21 standard.

RCTs, randomized controlled trials.

To evaluate the possible effect modification, the *Cochrane Review* performed several population‐level subgroup analyses, including analyses that stratify, on the basis of the study‐specific averages of maternal body mass index (BMI), maternal height, timing of supplementation, and the iron dose. Among the 10 trials in which the average maternal BMI was at least 20 kg/m^2^, there was evidence of a lower incidence of SGA among those who received MMS compared with IFA, while there was no evidence of a difference among the three trials where the mean BMI was less than 20 kg/m^2^ (*P* value for subgroup differences = 0.001). Similarly, among the six trials in which the average maternal height was at least 154.9 cm, MMS was associated with a reduction in SGA, while no effect was apparent in the eight trials in which the average maternal height was less than 154.9 cm (*P* value for subgroup differences <0.0001). Thus, while the review suggests that MMS reduces the risk of SGA, the reviewers caution that this effect was only observed in populations with better nutritional status, as defined by a height of at least 154.9 cm or a BMI of at least 20 kg/m^2^. However, among trials in which the average maternal BMI was less than 20 kg/m^2^, those receiving MMS had a lower rate of preterm birth, whereas no difference for preterm birth was observed among trials in which the average BMI was greater than or equal to 20 kg/m^2^ (*P* value for subgroup differences <0.0001). The authors found no subgroup differences by the dose of iron with regard to preterm birth, SGA, or perinatal mortality, on the basis of the 15 studies included in this subgroup analysis.

### Overview of the 2017 IPD meta‐analysis

Smith *et al*. obtained IPD from 17 randomized controlled trials—including 112,952 pregnancies—that compared the use of MMS, which included IFA, versus IFA alone.^4^ Fifteen of these trials were also included in the 2019 *Cochrane Review*. The IPD meta‐analysis found that MMS reduces the risk of stillbirth (on the basis of fixed effects analysis), very LBW (VLBW), LBW, early preterm birth, preterm birth, and SGA (by INTERGROWTH‐21 standards and Oken reference) when compared with IFA. This is in contrast to the *Cochrane Review*, which only found the evidence of effects of MMS on LBW and SGA. Table 2 contains a summary of these results in comparison to the results from the *Cochrane Review*. In addition, Smith *et al*. also found the evidence of increased risk of large for gestational age (LGA) (by Intergrowth standards but not by Oken reference), which was not examined in the *Cochrane Review*. While this may raise concerns about increased risk of obstructed labor/asphyxiation, the authors noted that MMS was not associated with increased risk of stillbirth or mortality at any time point, including among women with short stature (less than 150 cm), who are more likely to be at risk of obstructed labor.

Twenty‐six subgroup analyses were conducted with numerous outcomes in the IPD meta‐analysis to identify individual characteristics that may modify the effect of MMS as compared with IFA alone. These analyses (summarized in Box 1) revealed the evidence of larger benefits of MMS among women who were anemic (defined as hemoglobin <110 g/L for pregnant women), were underweight (BMI <18.5 kg/m^2^), started supplementation before 20 weeks of gestation, had higher supplement adherence (>95%), were carrying a female fetus, and had a skilled birth attendant.

**Box 1. Summary of effect modifiers^4^**

- The effects of MMS compared with IFA appeared greater among anemic pregnant women than nonanemic women for LBW (19% reduction for anemic women versus 9% reduction for nonanemic women; *P* value for heterogeneity 0.049), SGA (8% versus 1% reduction; *P* value for heterogeneity 0.03), and 6‐month infant mortality (29% versus 7% reduction; *P* value for heterogeneity 0.04).
- The effects of MMS compared with IFA appeared greater among underweight pregnant women than nonunderweight women in reducing the risk of preterm birth (16% for underweight women versus 6% for nonunderweight women; *P* value for heterogeneity 0.01).
- The effects of maternal MMS compared with IFA appeared greater among female infants than male infants with respect to reducing the risk of neonatal mortality (15% reduction for females versus 6% increase for males; *P* value for heterogeneity 0.007), 6‐month mortality (15% reduction for females versus 2% reduction for males; *P* value 0.06), and infant mortality (13% reduction for females versus 5% increase for males; *P* value for heterogeneity 0.04).
- The effects of MMS compared with IFA appeared greater among women who started supplementation before 20 weeks of gestation than those who started after 20 weeks in reducing the risk of preterm birth (11% reduction before 20 weeks versus no change after 20 weeks; *P* value for heterogeneity 0.03).
- The effects of MMS compared with IFA appeared greater among women who started supplementation after 20 weeks of gestation than those who started before 20 weeks in reducing the risk of stillbirth (19% reduction after 20 weeks versus 3% reduction before 20 weeks; *P* value for heterogeneity 0.05).
- The effects of MMS compared with IFA appeared greater when adherence was greater than or equal to 95% versus less than 95% for survival and birth outcomes, specifically for neonatal mortality (12% reduction for greater than or equal to 95% adherence versus 5% increase for less than 95% adherence; *P* value for heterogeneity 0.05) and infant mortality (15% reduction versus 6% increase; *P* value for heterogeneity 0.02).
- MMS did not significantly increase the risk of stillbirth, neonatal, 6‐month, or infant mortality in any of the 26 subgroups analyzed compared with IFA.

## Revisiting the risks and concerns of antenatal MMS

### Risk of neonatal mortality

**Figure 4:**
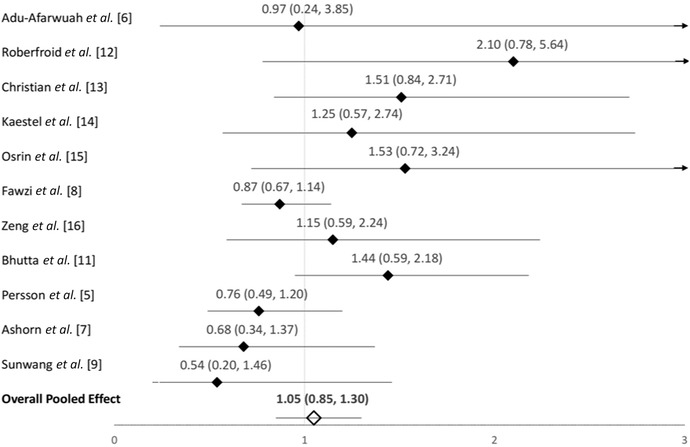
Forest plot for the effect of MMS versus IFA (with 60 mg of iron and any dose of folic acid) in the control group on neonatal mortality. This includes all available trials that included a 60 mg iron control group. Reproduced from Sudfeld and Smith.^44^

The WHO ANC Guidelines state that a source of concern in regard to recommending MMS was the potential risk of increased neonatal mortality.^42^ This concern arose from a meta‐analysis of a subset of trials performed for the WHO guidelines review (Fig. 10b of the subgroup analyses grouping trials according to the dose of iron in the control group in the WHO ANC Guidelines Web Supplement). The trials included in this subgroup analysis were (1) trials using MMS (containing any dose of IFA) compared with an IFA control group that received 60 mg of iron and 400 μg of folic acid and (2) trials using MMS (containing any dose of IFA) compared with an IFA control group that received 30 mg of iron and 400 μg of folic acid.^42^ Among trials using a 60 mg iron plus 400 μg of folic acid in the control group, the relative risk of neonatal mortality was 1.22 (0.95, 1.57) for those receiving MMS versus IFA in the subset of discordant iron doses.^42^ However, in these calculations, the effect estimate used for the Bhutta 2009 trial was incorrectly quoted, using a risk ratio of 0.97 (0.66, 1.45), which should have been 1.44 (0.99, 2.18).^43^ A recent reanalysis of these data, performed by Sudfeld and Smith, used the corrected risk ratio estimates of the Bhutta 2009 trial and five additional trials that provided 60 mg of iron in the IFA group.^44^ These five additional trials include the MINIMat trial group using a 60 mg iron dose (with 400 μg of folic acid) in the control arm,^45^ two trials with 60 mg of iron but only 250 μg of folic acid in the control group,^46^, ^47^ and two newer trials that were not previously available (lipid‐based supplement arms excluded).^48^, ^49^ With the inclusion of these studies and the updated risk ratio for the Bhutta trial, the risk ratio for neonatal mortality of MMS compared with IFA was 1.05 (0.85, 1.30) (Fig. 4).

### Risk of reaching the UL

The United Nations International Multiple Micronutrient Antenatal Preparation, or UNIMMAP, is the supplement most commonly used in MMS in trials with pregnant women. It provides 15 vitamins and minerals, including IFA. The composition of UNIMMAP largely reflects recommended dietary allowances (RDAs) or adequate intakes (AIs) (as of 1998) for each included vitamin and mineral, with some slightly lower dosages for folic acid and iodine.^50^, ^51^ Gernand modeled the impact of supplementation with UNIMMAP on the micronutrient intakes of pregnant women consuming a nutrient‐rich, diverse diet that meets the dietary intake recommendations from both WHO^52^ and the Institute of Medicine^53^ and found very little risk of excessive intakes (i.e., exceeding the upper level (UL) of recommended nutrient intakes). When UNIMMAP was provided along with a nutrient‐rich diet, intakes of iron, niacin, and folic acid reached or slightly exceeded the UL; but these were unlikely to result in significant adverse effects.^54^ More detailed information on the risk of exceeding the UL with MMS can also be found in Gernand.^54^ In regard to iron intake, the current WHO recommendation is 30–60 mg in pregnancy; however, the U.S. Institute of Medicine set the UL at 45 mg, suggesting that 30 mg may have a better safety margin than 60 mg.^51^

### Side effects and adherence data for MMS and IFA

Side effects and adherence to supplementation are important considerations for any supplementation program, as these could influence the effectiveness of the intervention. Unfortunately, these outcomes are not consistently reported in many trials comparing MMS and IFA, and differences in how outcomes are reported (e.g., number of times medical care was sought versus self‐reporting of specific symptoms) make it difficult to compare findings. Nonetheless, there does not appear to be any important difference in side effects between IFA and MMS in six of the seven trials that reported on side effects.^43^, ^55^, ^56^, ^57^, ^58^, ^59^ In a seventh trial, more women reported vomiting in the MMS group compared with the IFA group (MMS, 11.6%; IFA (60 mg iron), 6.9% (*P* = 0.002); and IFA (30 mg iron), 7.1% (*P* = 0.003)), but no evidence of differences was noted for any other side effect.^45^

Similarly, there were large differences in the methods used to assess adherence to the supplements (e.g., pills missing from bottles or self‐reported dosing), and not all studies reported on adherence. Despite these inconsistencies in reporting, differences in adherence rates between the IFA and MMS groups were generally very small, with no more than a 2% difference between the intervention groups in 10 trials that reported on adherence.^43^, ^48^, ^49^, ^55^, ^56^, ^57^, ^58^, ^59^, ^60^ Importantly, there were no trends toward better adherence to one supplement versus the other. On the basis of these available data, there appears to be no evidence of differences in self‐reported side effects or adherence between IFA and MMS.

A complicating factor with regard to analyzing data from trials that compared MMS with IFA is that the iron dose in the IFA control group was often greater than that in the MMS. For example, UNIMMAP was the most commonly used multiple micronutrient supplement formulation (eight trials), and it contains 30 mg of iron. In most of those eight trials, it was compared with an IFA supplement containing 60 mg of iron. Strictly speaking, this precludes knowing whether the effects of the MMS were attributable to the lower dose of iron versus the addition of other nutrients. The iron content in UNIMMAP was set at 30 mg to be a single RDA^51^ for pregnant women (27 mg is the current RDA^61^) similar to all other nutrients, and to avoid the risk of side effects because the presence of other micronutrients (e.g., vitamin A, vitamin C, and riboflavin) was expected to improve the absorption or utilization of iron compared with the iron in IFA alone. Unfortunately, there are very few trials that compared the effect on birth outcomes by dose of iron supplementation. The MINIMat trial in Bangladesh is the only study that used two IFA arms, one containing 60 mg iron and the other 30 mg, plus a multiple micronutrient supplement arm with 30 mg of iron. When comparing the two iron doses within the IFA treatment arms, there was no evidence of differences between 30 and 60 mg iron with regard to birthweight, anemia at 30 weeks’ gestation, stillbirth, early neonatal, perinatal, neonatal, and infant mortality rates. However, the study was powered for detecting differences in anemia and birthweight, not mortality.^45^

### Cost‐effectiveness

A narrowly focused cost‐effectiveness analysis of switching from IFA to MMS (single‐year, complete and immediate switching) has been performed for two countries, Bangladesh and Burkina Faso.^62^ For this analysis, they assumed a dose of 180 tablets per pregnant woman, which would cost an additional ∼US$0.88 for each pregnancy, and estimated the current coverage of IFA programs in each country. The results of the IPD meta‐analysis were used for the birth outcome data, using the overall effects and subgroup analyses.^4^ Making the switch to MMS in Bangladesh, where coverage of the national IFA program is estimated to be ∼50%, would cost approximately $39 per case of LBW averted, $85 per case of preterm birth averted, $62–$104 per case of SGA averted (INTERGROWTH‐21st and Oken standards, respectively), and $239 per case of infant mortality averted. Combining the years of life lost and disability‐adjusted life years (DALY) from sequelae of preterm birth, the cost per disability‐adjusted life year averted was $13.25 for calculations using overall effects and $4.60 for calculations accounting for different effects among population subgroups from the IPD meta‐analysis of MMS versus IFA on mortality and birth outcomes.^4^

In the case of Burkina Faso, where ∼10% of women reported taking at least 180 IFA tablets during their last pregnancy, authors estimated the following approximate costs: $58 per case of LBW averted, $102 per case of preterm birth averted, $91–$152 per case of SGA averted (INTERGROWTH‐21st and Oken standards, respectively), and $148 per case of infant mortality averted. Combining the years of life lost and disability‐adjusted life years from sequelae of preterm birth, the cost per DALY averted was $15.21 for calculations using overall effects and $3.56 for calculations accounting for effect modification in the effects of MMS versus IFA on mortality and birth outcomes. Assuming a cost differential of $2 per covered pregnancy (i.e., $3 per dose of the UNIMMAP formulation and $1 per dose of IFA; WHO, 2016) would approximately double these cost‐effectiveness estimates (increase of $2/$0.88 = ∼2.3).

Similar to existing IFA programs, programmatic inefficiencies and low adherence will increase the cost per outcome averted and dramatically so under some sets of assumptions. Nonetheless, the values reported above for multiple micronutrient supplementation are still likely to compare favorably to those of more complex interventions to prevent preterm birth and infant mortality, such as screening for and managing chronic disease among pregnant women.^62^ For example, pneumococcus and rotavirus vaccines and mother's groups to improve maternal/neonatal health were each estimated to cost more than $100 per DALY averted.^63^ Cost‐effectiveness of replacing IFA with MMS will be most favorable in countries with well‐performing IFA distribution programs.

It is also important to note that the cost‐effectiveness analyses undertaken in support of the task force and reported in this volume ignore programmatic transition costs and are based *exclusively* on the differences in IFA and multiple micronutrient tablet costs, which can be significant. However, increasing and sustained demand for multiple micronutrient tablets will reduce tablet prices owing to scale economies in production and in the procurement of multiple micronutrient tablet ingredients. Future work should expand the temporal focus of analysis (e.g., to 10 years) and include all of the transition costs and time lags associated with switching from IFA to MMS, which will likely vary by country. Future work should also assess (perhaps in the context of sensitivity analyses) the effects on benefits and on costs of consuming fewer than, and in excess of, the prescribed 180 tablets per pregnancy.

## Task force conclusions

The task force was convened to reassess the evidence base for antenatal MMS and to help policymakers understand under what circumstances and contexts MMS could be considered under the provision provided in the WHO ANC guideline stating that “policymakers in populations with a high prevalence of nutritional deficiencies might consider the benefits of MMN supplements on maternal health.” To describe these circumstances and contexts, the group also compiled and evaluated evidence regarding the prevalence of micronutrient deficiencies and adverse pregnancy outcomes. The new evidence confirms that inadequate micronutrient intakes, micronutrient deficiencies, adverse pregnancy outcomes, and infant mortality are still highly prevalent in LMICs.

Despite the variability in prevalence of micronutrient deficiencies among WRA and a general lack of data on dietary intake and micronutrient status among pregnant women in most LMICs, there is clear and consistent evidence from trial data that MMS may be beneficial in LMICs where micronutrient deficiencies are relatively common and would reduce the risk for preterm birth, SGA, and LBW.

In addition to the overall benefits to pregnant women and their infants, the IPD subgroup analyses revealed a greater potential to respond to MMS among anemic women, who had a reduced risk of LBW (19%), SGA (8%), and 6‐month mortality (29%) compared with the IFA group.^4^ In addition, female infants born to mothers receiving MMS had a significant reduction in neonatal (15%), 6‐month (15%), and infant mortality (13%) compared with female infants from mothers receiving IFA. Although results from post‐hoc subgroup analyses should be critically interpreted, the task force concludes that these results suggest that MMS might have the potential to further reduce the risk of these adverse outcomes among these subgroups.

It is important to note that these risk reductions for preterm birth, LBW, SGA, and stillbirth associated with MMS during pregnancy are in addition to the benefits provided by IFA supplements alone.^5^, ^64^ Additionally, these trials were performed across diverse settings in LMICs, where micronutrient deficiencies and adverse birth outcomes are relatively common. Thus, the task force concluded that these trials provide broad applicability to many settings in LMICs.

A cost‐effectiveness analysis was performed on the basis of the data provided by all trials and the subset that used equal doses of iron included in the IPD meta‐analysis.^62^ The cost per multiple micronutrient tablet will be slightly higher than that of IFA, and the very large volume of tablets required to cover all pregnancies will cause even small differences in the multiple micronutrient supplement versus IFA tablet costs to translate into large outlays. However, the task force expects the additional mortality and birth outcome benefits associated with the more expensive multiple micronutrient tablets to generally result in favorable cost‐effectiveness values when compared with other programs aimed at reducing mortality and undesirable birth outcomes, as described by Engle‐Stone *et al*.^62^ In addition, when produced and purchased at scale, the cost differential between IFA and multiple micronutrient tablets would be further reduced, which would further improve the cost‐effectiveness of multiple micronutrient supplementation programs. That said, the cost‐effectiveness of multiple micronutrient supplementation programs will likely be reduced in countries in which IFA programs perform poorly or when considering the country‐specific planning, implementation, monitoring, and evaluation costs associated with transitioning from IFA to multiple micronutrient tablets. These additional costs were not addressed in the analyses considered by the task force nor were the timeframes for these transitions considered.

The UNIMMAP formulation was specifically designed to meet the increased micronutrient requirements of pregnancy.^50^ The supplement contains 15 micronutrients at or near the levels needed during pregnancy, including 30 mg of iron, which is within the 30–60 mg of daily iron recommended by the WHO. Despite the levels of micronutrients near the RDA or AI, there is little risk of exceeding the UL of micronutrients even when combined with adequate dietary intake. In addition, the trials that reported on side effects and adherence showed no difference in adherence and generally no differences in reported side effects between MMS and IFA, suggesting that compliance to these supplements will be similar.^4^ On the basis of the use of the UNIMMAP formulation in many of the trials, the task force concluded that this formulation should be used for antenatal supplementation with MMS going forward. There is evidence of greater benefit when started early in pregnancy and should be continued for the duration of the pregnancy. In many contexts, this equates to approximately 6 months (or 180 days).

On the basis of this review of evidence, the task force concluded that the use of a daily MMS does not increase the risk of adverse effects, has a number of additional benefits for mortality and birth outcomes compared with IFA, and can be a cost‐effective intervention for pregnant women in LMICs, where multiple micronutrient deficiencies persist. To identify the settings where supplementation with MMS is most likely to benefit pregnant women and their infants, the task force suggests using available biomarker, dietary intake, or diet diversity data to determine the prevalence of multiple micronutrient deficiencies or estimate the likelihood of deficiencies. Other indicators, such as the prevalence of low maternal BMI, maternal anemia, LBW, SGA, and preterm births, may also be used as complementary data. Where these conditions are prevalent, multiple micronutrient supplementation is likely to lead to additional benefits as compared with those attributable to IFA supplementation alone, and could be included as part of routine ANC to improve maternal micronutrient status and to reduce the risks of mortality and adverse birth outcomes.

## Disclaimer

R.K., A.F., and E.M. are UNICEF staff members. The opinions and statements in this article are those of the authors and may not reflect official UNICEF policies.

## Supporting information

Appendix 1 – Task Force Members

Appendix 2 – Micronutrient Deficiency Prevalence Review Search Strategy and Results

**Table S1**. Vitamin deficiencies in women of reproductive age (WRA) in low‐ and middle‐income countries (LMICs)
**Table S2**. Mineral deficiencies in women of reproductive age (WRA) in low‐ and middle‐income countries (LMICs)
**Table S3**. Anemia in pregnant women in low‐ and middle‐income countries (LMICs)

**Table S4**. Vitamin deficiencies among pregnant women in low‐ and middle‐income countries (LMICs)
**Table S5**. Mineral deficiencies among pregnant women in low‐ and middle‐income countries (LMICs)
