# Supplementary material for: Review of the evidence regarding the use of antenatal multiple micronutrient supplementation in low‐ and middle‐income countries
Source: Ann N Y Acad Sci. 2019 May 27;1444(1):6–21. doi: 10.1111/nyas.14121 (PMC6852202; doi:10.1111/nyas.14121)
Supplement: Supplementary file 1 — Appendix 1 – Task Force Members [file NYAS-1444-6-s001.docx]

# Appendix 1 – Task Force Members

Seth Adu-Afarwuah

Clayton Ajello

Lindsay Allen

Gilles Bergeron

Robert Black

Megan W. Bourassa

Parul Christian

Simon Cousens

Saskia de Pee

Luz Maria De Regil

Kathryn G. Dewey

Shams El Arifeen

Reina Engle-Stone

Alison Fleet

Alison D. Gernand

John Hoddinott

Rolf Klemm

Klaus Kraemer

Roland Kupka

Erin McLean

Habibe Millat

Sophie E. Moore

Lynnette M. Neufeld

Banda Ndiaye

Saskia J.M. Osendarp

Lars-Åke Persson

Kathleen M. Rasmussen

Anuraj H. Shankar

Emily Smith

Christopher R. Sudfeld

Emorn Udomkesmalee

Steve Vosti
