# Supplementary material for: Review of the evidence regarding the use of antenatal multiple micronutrient supplementation in low‐ and middle‐income countries
Source: Ann N Y Acad Sci. 2019 May 27;1444(1):6–21. doi: 10.1111/nyas.14121 (PMC6852202; doi:10.1111/nyas.14121)
Supplement: Supplementary file 2 — Appendix 2 – Micronutrient Deficiency Prevalence Review Search Strategy and Results [file NYAS-1444-6-s002.docx]

# Appendix 2 – Micronutrient Deficiency Prevalence Review Search Strategy and Results

An update of the Black et al. 2011 review was performed recently including a search of reviews published from 2013 until July 2017 in PubMed using the following search strategy: (woman OR women OR female OR maternal OR pregnant* OR prenatal OR reproductive age) AND (micronutrient OR trace element*) with the specific terms, as follows: “vitamin A”, “vitamin B1 OR thiamin*”, “vitamin B2 OR riboflavin*”, “vitamin B3 OR niacin*”, “vitamin B6 OR pyridox*”, “folic acid OR folate”, “vitamin B12 OR *cobalamin*”, “vitamin C”, “vitamin D”, “vitamin E OR *Tocopherol*”, “zinc”, “iodine”, “iron”, “copper”, “selenium”, “vitamin K”, “calcium”, or “phosphorus”). In addition, the most recent national surveys with available micronutrient data were obtained through personal contacts at University of California, Davis; International Zinc Nutrition Consultative Group (IZiNCG); and the World Health Organization (WHO). A total of 52 national or regional surveys and 29 reviews were identified, published after 2013 that reported on micronutrient status in pregnant women or women of reproductive age. With the exception of iodine and anemia, prevalence data on maternal micronutrient deficiencies were lacking for many countries, and the review therefore focused on estimating prevalence of micronutrient deficiencies in WRA. Weighted regional estimates were calculated from these 52 national or regional surveys when data on micronutrient status were available for three or more countries of a WHO region. Estimates for Iron Deficiency Anemia (IDA) in pregnant women were estimated from anemia data assuming that 50% of anemia is caused by iron deficiency [7].

The studies used different cut-offs to define deficiencies and detailed information on the cut-offs used per survey can be found in the Appendix 3 and 4. Vitamin A deficiency was defined as serum retinol <0.7 µmol/L (<20 µg/dL), <1.05 µmol/L or Retinol Binding Protein (RBP) <0.7 µmol/L or <1.05 µmol/L. Vitamin B-2 (Riboflavin) deficiency was defined as <11.3 nmol/L, while vitamin B-6 (pyridoxine) deficiency was defined as Pyridoxal-5’-phosphate, <19 nmol/L. Vitamin B-9 (folate) deficiency was defined as serum/plasma folate <10 nmol/L, <6.7 or 6.8 nmol/L (<3 ng /mL) or RBC <340 nmol/L (151 ng/mL) and vitamin B-12 as <148 pmol/L, <150 pmol/L (<203 pg/mL), <200 pmol/L, or <210 pmol/L. Vitamin D has various cut-offs among surveys: serum <50 nmol/L (≤20 ng/mL), <30 nmol/L (≤12 ng/mL), <25 nmol/L, or < 18 nmol/L. Whereas for vitamin E deficiency, cut-offs for α-tocopherol used were <5 µg/dL, <9.3 µmol/L and <12 µmol/L.

For iodine, all studies included in the review used median UIC <100 µg/L to define iodine deficiency in WRA and the cut-off for pregnant women was <150 µg/L. For zinc, the most common cut-off used were the IZiNCG cut-offs: <70 µg /dL for fasting adults in the morning and <66 µg /dL for nonfasting adults in the morning, and <59µg /dL for nonfasting adults in the afternoon [14]. But other zinc-cut offs were also reported: plasma/serum <80 µg/dLdL, <66 µg/dL, <65 µg/dL, <60µg/dL, <50 µg/dL (<7.65 µmol/L), <56.2 µg/dL (<8.6 µmol/L), <10 mmol/L, or <65 µmol/L (425 µg/dL). For iron deficiency, the biomarker was serum ferritin with almost all reports using the cut-off of <15 µg/L and only 4 studies used <12 ng/mL (<12 µg/L) and 1 study used 11 µg/L. To define anemia, all hemoglobin thresholds were <120 g/L or <12 g/dL except the study in Timor Leste which used 11 g/dL, while studies in pregnant women used the cut-off <110 g/L (<11 g/dL). Selenium and calcium deficiencies were defined as plasma/serum <0.75 µmol/L and <8.4 mg/dL, respectively.
