# Supplementary material for: Review of the evidence regarding the use of antenatal multiple micronutrient supplementation in low‐ and middle‐income countries
Source: Ann N Y Acad Sci. 2019 May 27;1444(1):6–21. doi: 10.1111/nyas.14121 (PMC6852202; doi:10.1111/nyas.14121)
Supplement: Supplementary file 3 — Table S1. Vitamin deficiencies in women of reproductive age (WRA) in low‐ and middle‐income countries (LMICs) Table S2. Mineral deficiencies in women of reproductive age (WRA) in low‐ and middle‐income countries (LMICs) Table S3. Anemia in pregnant women in low‐ and middle‐income countries (LMICs) [file NYAS-1444-6-s003.docx]

**Prevalence of micronutrient deficiencies in Women of Reproductive Age (WRA)**

**Table S1. Vitamin deficiencies in Women of Reproductive Age (WRA) in Low- and Middle-Income Countries (LMIC)**

| **Region** | **First author, publication year** | **Country, year of field study** | **N** | **Setting of study population** | **Biomarker, vitamin cut-off** | **Vitamin deficiency, %** | **Vitamin deficiency (n)** |
| --- | --- | --- | --- | --- | --- | --- | --- |
| **Vitamin A** | | | | | | | |
| AFR | Ethiopian Public Health Institute, 2016 [1] | Ethiopia, 2015 | 1619 | National | Serum retinol, <0.70 µmol/l | 3.4 | 55 |
|  | Alaofe, 2017 [2] | Benin, 2014 | 632 | Local, Kalale District | Corrected serum retinol, <20 µg/dl | 17.72 | 112 |
|  | Liberia Institute of Statistics, 2011 [3] | Liberia, 2011 | 2240 | National | Serum retinol, <0.70 µmol/l | 2.2 | 49 |
|  | Cameroon MoPH, 2009 [4] | Cameroon, 2009 | 689 | National | RBP, <0.7 µmol/L | 2.2 | 15 |
|  | Wirth, 2017 [5] | Cote d’lvoire, 2007 | 850 | National, BRINDA Project | RBP or retinol  concentration, <1.05 µmol/L^a^ | 13.2 | 112 |
|  | International Institute of Tropical Agriculture, 2004^c^ [6] | Nigeria, 2001-2003 | 3,148 | National | Serum retinol <20 µg/dl | 4.1 | 129 |
| SEAR | Timor-Leste Ministry of Health, 2015 [7] | Timor-Leste, 2013 | 616 | National | Serum retinol, <1.05 µmol/L | 13.5 | 83 |
|  | International Centre for Diarrheal Diseases Research, 2013^d^ [8] | Bangladesh, 2011-2012 | 918 | National | Serum retinol, <0.70 µmol/l | 5.4 | 50 |
|  | Madanijah, 2016^e^ [9] | Indonesia, 2010-2011 | 45 | Local, Bogor District | Serum retinol, <200 µg/l | 6.7 | 3 |
| EUR | Kyrgyzstan Ministry of Health, 2009^c^ [10] | Kyrgyztan, 2009 | 1138 | National | RBP, <0.7 µmol/l | 0.6 | 7 |
| EMR | Ministry of Public Health, 2013 [11] | Afghanistan, 2013 | 1187 | National | Serum retinol, <0.70 µmol/l | 11.3 | 134 |
|  | Aga Khan University, 2011^e^ [12] | Pakistan, 2011 | 6925 | National | Serum retinol, <0.70 µmol/l | 43.2 | 2992 |
|  | Ministry of Health, 2010 [13] | Jordan, 2010 | 2032 | National | Serum retinol, <0.70 µmol/l | 4.8 | 98 |
| WPR | National Institute of Statistic, 2015^c^ [14] | Cambodia, 2014 | 739 | National | RBP <1.05 µmol/l | 8.7 | 64 |
|  | Laillou, 2012 [15] | Vietnam, 2010 | 1475 | National | Serum retinol, <0.70 µmol/l | 1.6 | 24 |
|  | Wirth, 2017 [5] | Papua New Guinea, 2005 | 749 | National | RBP or retinol  concentration, <1.05 µmol/l^a^ | 7.7 | 58 |
| **Vitamin B-9 (Folate)** | | | | | | | |
| AFR | Ethiopian Public Health Institute, 2016 [1] | Ethiopia, 2015 | 1647 | National | Serum, <6.8 nmol/L | 17.3 | 285 |
|  | Cameroon MoPH, 2009 [4] | Cameroon, 2009 | 391 | National | Serum, <10 nmol/L | 16.6 | 65 |
|  | Wirth, 2017 [5] | Cote d’lvoire, 2007 | 850 | National, BRINDA Project | Plasma/Serum, <10 nmol/L. | 86.4 | 735 |
| AMR | MSPAS, 2012 [16] | Guatemala, 2009-2010 | 1448 | National | Serum, <3 ng / mL | 0.7 | 10 |
| SEAR | International Centre for Diarrheal Diseases Research, 2013 [8] | Bangladesh, 2011-2012 | 849 | National | Plasma, <6.8 nmol/L | 9.1 | 78 |
| EUR | Kyrgyzstan Ministry of Health, 2009^c^[10] | Kyrgyztan, 2009 | 1325 | National | RBC folate, < 151 ng/mL | 37.4 | 496 |
|  | UNICEF, 2010 [17] | Georgia, 2009 | 407 | National | Serum, <10 nmol/L | 36.6 | 149 |
|  | Clewes, 2012 [18] | Uzbekistan, 2008 | 2582 | National | Serum, <10 nmol/L | 28.8 | 744 |
| EMR | Tawfik, 2014 [19] | Egypt, N/R | 579 | National | Serum, <10 nmol/L | 14.7 | 85 |
|  | Ministry of Health, 2010 [13] | Jordan, 2010 | 393 | National | RBC folate, <151 ng/mL | 13.6 | 54 |
| WPR | National Institute of Statistic, 2015 [14] | Cambodia, 2014 | 739 | National | Serum, <10 nmol/L | 19.2 | 142 |
|  | Laillou, 2012 [15] | Vietnam, 2010 | 1472 | National | Plasma, <6.8 nmol/L | 2.7 | 40 |
| **Vitamin B-12 (Cobalamin)** | | | | | | | |
| AFR | Ethiopian Public Health Institute, 2016 [1] | Ethiopia, 2015 | 1619 | National | Plasma, <203 pg/ml | 15.1 | 245 |
|  | Cameroon MoPH, 2009 [4] | Cameroon, 2009 | 391 | National | Plasma <210 pmol/L | 28.6 | 112 |
|  | Wirth, 2017 [5] | Cote d’lvoire, 2007 | 850 | National, BRINDA Project | Plasma, <150 pmol/L | 18.0 | 153 |
| AMR | MSPAS, 2012 [16] | Guatemala, 2009-2010 | 1448 | National | Plasma <148 pmol/L | 18.9 | 274 |
| SEAR | International Centre for Diarrheal Diseases Research, 2013 [8] | Bangladesh, 2011-2012 | 872 | National | Plasma, <200 pmol/L | 6.1 | 53 |
| EMR | Ministry of Health, 2010 [13] | Jordan, 2010 | 2039 | National | Plasma, <200 pg/ml | 11.1 | 226 |
| WPR | National Institute of Statistic, 2015 [14] | Cambodia, 2014 | 739 | National | Plasma, <150 pmol/L | 1.1 | 8 |
|  | Laillou, 2012 [15] | Vietnam, 2010 | 505 | National | Plasma, <148 pmol/l, | 11.7 | 59 |
| **Vitamin D** | | | | | | | |
| SEAR | Bromage, 2016^d^ [20] | Bangladesh, 2011-2012 | 631 | National | Serum, <50 nmol/l | 71.5 | 451 |
| EMR | Ministry of Public Health, 2013 [11] | Afghanistan, 2013 | 1190 | National | Serum, ≤20 ng/ml | 95.5 | 1137 |
|  | Aga Khan University, 2011^c [12]^ | Pakistan, 2011 | 5402 | National | Serum, ≤20 ng/ml | 66.2 | 3576 |
|  | Ministry of Health, 2010 [13] | Jordan, 2010 | 2032 | National | Serum, ≤12 ng/ml | 60.3 | 1225 |
| WPR | Uush, 2013 [21] | Mongolia, 2010 | 867 | National | Serum, < 18 nmol/L | 30 | 260 |
|  | Smith, 2016 [22] | Cambodia, 2016 | 725 | National | Serum, <50 nmol/l | 29 | 210 |

N/R: Not reported; AFR: Africa; AMR: America; SEAR: South East Asia; EUR: Europe; EMR: Eastern Mediterranean; WPR: Western Pacific

1. % of insufficiency; b. Mothers/Non-pregnant mothers; c. Non-pregnant Non-lactating women; d. Pre-pregnant

**Table S2. Mineral deficiencies in Women of Reproductive Age (WRA) in Low- and Middle-Income Countries (LMIC)**

| **Region** | **First author, publication year** | **Country, year of field study** | **N** | **Setting of study population** | **Biomarker, Mineral cut-off** | **Mineral deficiency, %** | **Mineral deficiency, n** |
| --- | --- | --- | --- | --- | --- | --- | --- |
| **Iodine*** |  |  |  |  |  |  |  |
| AFR | Ethiopian Public Health Institute, 2016 [1] | Ethiopia, 2015 | 1707 | National | UIC, <100 µg/l | 51.8 | 884 |
|  | UNICEF, 2009^a^[23] | Malawi, 2009 | 529 | National | UIC, <100 µg/l | 25.7 | 136 |
|  | FSNAU, 2009[24] | Somalia, 2009 | 1919 | National | UIC, <100 µg/l | 13.8 | 265 |
|  | International Institute of Tropical Agriculture, 2004^b^ [6] | Nigeria, 2001-2003 | 3104 | National | UIC, <100 µg/l | 30.7 | 953 |
| SEAR | Timor-Leste Ministry of Health, 2015 [7] | Timor-Leste, 2013 | 629 | National | UIC, <100 µg/l | 26.7 | 168 |
|  | International Centre for Diarrheal Diseases Research, 2013 [8] | Bangladesh, 2011-2012 | 1273 | National | UIC, <100 µg/l | 42.1 | 536 |
| EUR | Ministry of Health, 2009 [25] | Tajikistan, 2009 | 2137 | National | UIC, <100 µg/l | 58.6 | 1252 |
| EMR | Ministry of Public Health, 2013 [11] | Afghanistan, 2013 | 1135 | National | UIC, <100 µg/l | 40.7 | 462 |
| WPR | National Institute of Statistic, 2015 [14] | Cambodia, 2014 | 737 | National | UIC, <100 µg/l | 78 | 575 |
| **Zinc** |  |  |  |  |  |  |  |
| AFR | Ethiopian Public Health Institute, 2016 [1] | Ethiopia, 2015 | 1625 | National | IZiNCG cutoffs^a^ | 34 | 553 |
|  | Hess, 2017 [26] | Senegal, 2010 | 1082 | National | IZiNCG cutoffs^a^ | 59 | 638 |
|  | Engle-Stone, 2014 [27] | Cameroon, 2009 | 879 | National | Adjusted Plasma, <66 µg/dl | 81.6 | 717 |
|  | International Institute of Tropical Agriculture, 2004^b^ [6] | Nigeria, 2001-2003 | 3779 | National | Plasma, <80 µg/dl | 28.1 | 1062 |
|  | Hess, 2017 [26] | Kenya, 1999 | 1093 | National | Serum, <65 µg/dL | 52 | 568 |
| SEAR | International Centre for Diarrheal Diseases Research, 2013^c^ [8] | Bangladesh, 2011-2012 | 1073 | National | Serum, <10 mmol/L | 57.3 | 615 |
|  | Madanijah, 2016^d^ [9] | Indonesia, 2010-2011 | 45 | Local, Bogor District | Plasma, <65 µmol/l | 42.2 | 19 |
| EMR | Ministry of Public Health, 2013 [11] | Afghanistan, 2013 | 1187 | National | Serum, <60 µg/dl | 23.4 | 278 |
|  | Aga Khan University, 2011^c^ [12] | Pakistan, 2011 | 5953 | National | Serum, <60 µg/dl | 41.6 | 2476 |
| WPR | Wieringa, 2016 [28] | Cambodia, 2014 | 720 | National | Plasma, <7.65 µmol/L | 26.3 | 189 |
|  | Laillou, 2012 [15] | Vietnam, 2010 | 1522 | National | IZiNCG cutoffs^a^ | 67.2 | 1023 |
|  | Marcos, 2008^e^ [29] | Philippines, 2008 | 2892 | National | IZiNCG cutoffs^a^ | 31.2 | 902 |
| **Iron Deficiency** | | | | | | | |
| AFR | Ethiopian Public Health Institute, 2016 [1] | Ethiopia, 2015 | 1630 | National | Serum ferritin, <15 µg/l | 10 | 163 |
|  | Alaofe, 2017 [2] | Benin, 2014 | 609 | Local, Kalalé district | Serum ferritin with adjustment^a^ | 18.3 | 111 |
|  | Petry, 2016 [30] | Mozambique, 2012-2013 | 1068 | National | Serum ferritin, <15 µg/l | 25.1 | 268 |
|  | Petry, 2016 [30] | Sierra Leone, 2013 | 774 | National | Serum ferritin, <15 µg/l | 8.3 | 64 |
|  | Petry, 2016 [30] | Cameroon, 2012 | 872 | National | Serum ferritin, <15 µg/l | 15.3 | 133 |
|  | Petry, 2016 [30] | Kenya, 2011 | 633 | National | Serum ferritin, <15 µg/l | 21.3 | 135 |
|  | Liberia Institute of Statisticsm, 2011 [3] | Liberia, 2011 | 2240 | National | Serum ferritin, <15 µg/l | 19.6 | 439 |
|  | Petry, 2016 [30] | Côte d’Ivoire, 2007 | 910 | National | Serum ferritin, <15 µg/l | 16.7 | 152 |
| AMR | Petry, 2016 [30] | Nicaragua, 2003-2005^d^ | 1500 | National | Serum ferritin, <15 µg/l | 30.9 | 464 |
|  | MSPAS, 2012 [16] | Guatemala, 2009-2010 | 1448 | National | Serum ferritin <15.0 ng/ml | 18.4 | 267 |
| SEAR | Timor-Leste Ministry of Health, 2015 [7] | Timor-Leste, 2013 | 592 | National | Serum ferritin <15.0 ng/ml | 21.3 | 126 |
|  | International Centre for Diarrheal Diseases Research, 2013 [8] | Bangladesh, 2011-2012 | 882 | National | Serum ferritin <15.0 ng/ml | 7.1 | 63 |
|  | Madanijah, 2016^d^ [9] | Indonesia, 2010-2011 | 45 | Local, Bogor District | Serum ferritin, <15 µg/l | 23.1 | 10 |
| EUR | UNICEF, 2010 [17] | Georgia, 2009 | 472 | National | Serum ferritin, <15 µg/l | 1.6 | 19 |
|  | Petry, 2016 [30] | Tajikistan, 2009 | 2138 | National | Serum ferritin, <15 µg/l | 9.7 | 207 |
|  | Clewes, 2012 [18] | Uzbekistan, 2008 | 2582 | National | Serum ferritin, <15 µg/l | 47.5 | 1227 |
| EMR | Ministry of Public Health, 2013 [11] | Afghanistan, 2013 | 1187 | National | Serum ferritin <12ng/ml | 24 | 285 |
|  | Aga Khan University, 2011 [12] | Pakistan, 2011 | 7415 | National | Ferritin, <12ng/dL | 26.8 | 1987 |
| WPR | Wieringa, 2016 [28] | Cambodia, 2014 | 720 | National | Serum ferritin, <15 µg/l | 6.9 | 50 |
|  | Laillou, 2012 [15] | Vietnam, 2010 | 1523 | National | Serum ferritin, <15 µg/l | 13.7 | 209 |
|  | Petry, 2016 [30] | Mongolia, 2010 | 767 | National | Serum ferritin, <15 µg/l | 28.2 | 216 |
|  | Petry, 2016 [30] | Lao PDR, 2006 | 818 | National | Serum ferritin, <15 µg/l | 23.2 | 190 |
| **Iron Deficiency Anemia** | | | | | | | |
| AFR | Ethiopian Public Health Institute, 2016 [1] | Ethiopia, 2015 | 1532 | National | Hb, <120 g/l & Serum ferritin, <15 mg/l | 4.7 | 72 |
|  | Alaofe, 2017 [2] | Benin, 2014 | 609 | Local, Kalalé district | Hb, <120 g/L & Adjusted ferritin, <15 mg/l | 11.3 | 69 |
|  | Petry, 2016 [30] | Cameroon, 2012 | 857 | National | Anaemia & Serum ferritin <15µg/l | 11.1 | 95 |
|  | Petry, 2016 [30] | Mozambique, 2012-2013 | 1068 | National | Anaemia & Serum ferritin <15µg/l | 16.1 | 172 |
|  | Petry, 2016 [30] | Sierra Leone, 2013 | 827 | National | Anaemia & Serum ferritin <15µg/l | 6.1 | 50 |
|  | Liberia Institute of Statistics, 2011 [3] | Liberia, 2011 | 1911 | National | Hb, <12 g/dl & Serum ferritin <15µg/l | 11.3 | 216 |
|  | Petry, 2016 [30] | Kenya, 2011 | 592 | National | Anaemia & Serum ferritin <15µg/l | 14 | 83 |
|  | Petry, 2016 [30] | Côte d’Ivoire, 2007 | 905 | National | Anaemia & Serum ferritin <15µg/l | 11.6 | 105 |
| AMR | Petry, 2016 [30] | Nicaragua, 2003-2005^d^ | 1500 | National | Anaemia & Serum ferritin <15µg/l | 6.9 | 104 |
| SEAR | Timor-Leste Ministry of Health, 2015 [7] | Timor-Leste, 2013 | 580 | National | Hb, <11 g/dl & Serum ferritin <15µg/l | 15.7 | 91 |
|  | International Centre for Diarrheal Diseases Research, 2013 [8] | Bangladesh, 2011-2012 | 868 | National | Hb, <12 mg/dl & Serum Ferritin, <15 ng/ml | 4.8 | 42 |
| EUR | UNICEF, 2010 [17] | Georgia, 2009 | 237 | National | Anaemia & Serum ferritin <15µg/l | 3.8 | 9 |
|  | Petry, 2016 [30] | Tajikistan, 2009 | 2138 | National | Anaemia & Serum ferritin <15µg/l | 2.2 | 47 |
| EUR | Petry, 2016 [30] | Uzbekistan, 2008 | 2582 | National | Hb, <12 mg/dl & Serum ferritin <15µg/l | 21.8 | 563 |
| EMR | Ministry of Public Health, 2013 [11] | Afghanistan, 2013 | 1187 | National | Hb, <12 mg/dl & Serum ferritin <12 ng/ml | 13.8 | 164 |
|  | Aga Khan University, 2011 [12] | Pakistan, 2011 | 6873 | National | Hb, <12 mg/dl & Ferritin, <12ng/dl | 19.9 | 1368 |
| WPR | Petry, 2016 [30] | Cambodia, 2014 | 450 | National | Anaemia & Serum ferritin <15µg/l | 2.2 | 10 |
|  | Petry, 2016 [30] | Mongolia, 2010 | 767 | National | Anaemia & Serum ferritin <15µg/l | 3.0 | 23 |
|  | Laillou, 2012 [15] | Vietnam, 2010 | 1522 | National | Hb, ,120 g/l & Adjusted ferritin, 15 mg/l | 5.4 | 83 |
|  | Petry, 2016 [30] | Lao PDR, 2006 | 818 | National | Anaemia & Serum ferritin <15µg/l | 14.6 | 119 |
| **Anemia** |  |  |  |  |  |  |  |
| AFR | Ethiopian Public Health Institute, 2016 [1] | Ethiopia, 2015 | 1741 | National | Hb, <12 g/dl | 17.7 | 308 |
|  | Rwanda NISR, 2015 [31] | Rwanda, 2014-2015 | 4331 | National | Hb, <12 g/dl | 18.7 | 810 |
|  | Lesotho Ministry of Health, 2016 [32] | Lesotho, 2014 | 2676 | National | Hb, <12 g/dl | 27.3 | 731 |
|  | Ghana Statistical Service, 2015 [33, 34] | Ghana, 2014 | 3262 | National | Hb, <12 g/dl | 41.3 | 1347 |
|  | Ministère de la Planification, 2015 [34] | Togo, 2013-2014 | 3104 | National | Hb, <12 g/dl | 47.6 | 1478 |
|  | Alaofe, 2017 [2] | Benin, 2014 | 679 | Local, Kalalé district | Hb, <12 g/dl | 47.7 | 324 |
|  | National Institute of Statistics, 2014 [35] | Sao Tome and Principle, 2014 | 2526 | National | Hb, <12 g/dl | 45.7 | 1154 |
|  | The Namibia MoHSS, 2014 [36] | Namibia, 2013 | 3369 | National | Hb, <12 g/dl | 20 | 674 |
|  | MPSMRM, 2014 [37] | Democratic Republic of Congo, 2012-2013 | 4927 | National | Hb, <12 g/dl | 37.7 | 1858 |
|  | Petry, 2016 [30] | Mozambique, 2012-2013 | 1086 | National | N/R | 39.8 | 432 |
|  | Petry, 2016 [30] | Sierra Leone, 2013 | 871 | National | N/R | 44.8 | 390 |
|  | Institut National de la Statistique, 2013 [38] | Guinea, 2013 | 2797 | National | Hb, <12 g/dl | 45.1 | 1261 |
|  | Petry, 2016 [30] | Cameroon, 2012 | 888 | National | N/R | 38.8 | 345 |
|  | INSAE, 2013 [39] | Benin, 2011-2012 | 3228 | National | Hb, <12 g/dl | 40.8 | 1317 |
|  | Congo CNSEE, 2013 | Congo, 2011-2012 | 3660 | National | Hb, <12 g/dl | 54.5 | 1995 |
|  | INS, 2013 [40] | Niger, 2012 | 2254 | National | Hb, <12 g/dl | 44.4 | 1001 |
| AMR | MSPAS, 2017 [16] | Guatemala, 2014-2015 | 19545 | National | Hb, <12 g/dl | 12.3 | 2404 |
|  | Cayemittes, 2013 [41] | Haiti, 2012 | 7479 | National | Hb, <12 g/dl | 49.3 | 3687 |
|  | Honduras Secretaria de Salud, 2013 [42] | Honduras, 2012 | 17158 | National | Hb, <12 g/dl | 15 | 2574 |
|  | Petry, 2016 [30] | Nicaragua, 2003-2005 ^d^ | 1500 | National | N/R | 11.2 | 168 |
| SEAR | Ministry of Health and Sports, 2017 [43] | Myanmar, 2015-2016 | 10233 | National | Hb, <12 g/dl | 45.8 | 4687 |
|  | Bhutan Ministry of Health, 2015 [44] | Bhutan, 2015 | 5176 | National | Hb, <12 g/dl | 34.9 | 1806 |
|  | Timor-Leste Ministry of Health, 2015 [7] | Timor-Leste, 2013 | 628 | National | Hb, <11 g/dl | 38.2 | 240 |
|  | Nepal Ministry of Health and Population, 2013 [45] | Nepal, 2012 | 4447 | National | Hb, <12 g/dl | 33 | 1468 |
|  | International Centre for Diarrheal Diseases Research, 2013 [8] | Bangladesh, 2011-2012 | 1031 | National | Hb, <12 g/dl | 26 | 268 |
| EMR | Ministry of Health and Population, 2014 [46] | Egypt, 2014 | 4986 | National | Hb, <12 g/dl | 25 | 1247 |
|  | Ministry of Public Health, 2013 [11] | Afghanistan, 2013 | 1187 ^c^ | National | Hb, <12 g/dl | 40.4 | 480 |
|  | Department of Statistics, 2012 [47] | Jordan, 2012 | 10734 | National | Hb, <12 g/dl | 33.5 | 3596 |
| WPR | National Institute of Statistics, 2015 [14] | Cambodia, 2014 | 9106 | National | Hb, <12 g/dl | 43.8 | 3988 |

N/R: Not reported; *Median Urinary Iodine Concentration, except: Nigeria; AFR: Africa; AMR: America; SEAR: South East Asia; EUR: Europe; EMR: Eastern Mediterranean; WPR: Western Pacific

1. Women of child-bearing age; b. Mothers/Non-pregnant mothers; c. Non-pregnant Non-lactating women; d. Pre-pregnant; e. Adult female

**Table S3. Anemia in pregnant women in Low- and Middle-Income Countries (LMIC)**

| **Region** | **First author, publication year** | **Country, year of field study** | **N** | **Setting of study population** | **Cut-off** | **Anaemia, %** | **Anaemia, n** |
| --- | --- | --- | --- | --- | --- | --- | --- |
| AFR | Rwanda NISR, 2015 [31] | Rwanda, 2014-2015 | 491 | National | Hb, <11 g/dl | 23.4 | 114 |
|  | Lesotho Ministry of Health, 2016 [32] | Lesotho, 2014 | 137 | National | Hb, <11 g/dl | 35.5 | 49 |
|  | Ghana Statistical Service, 2015 [33] | Ghana, 2014 | 341 | National | Hb, <11 g/dl | 44.6 | 152 |
|  | Ministère de la Planification, 2015[34] | Togo, 2013-2014 | 427 | National | Hb, <11 g/dl | 64.1 | 274 |
|  | The Namibia MoHSS, 2014 [36] | Namibia, 2013 | 288 | National | Hb, <11 g/dl | 25.6 | 74 |
|  | MPSMRM, 2014 [37] | Democratic Republic of Congo, 2012-2013 | 1126 | National | Hb, <11 g/dl | 43.4 | 489 |
|  | National Institute of Statistics, 2014 [35] | Sao Tome and Principle, 2014 | 228 | National | Hb, <11 g/dl | 61.4 | 140 |
|  | INSAE, 2013 [39] | Benin, 2011-2012 | 484 | National | Hb, <11 g/dl | 42.2 | 204 |
|  | Congo CNSEE, 2013 [40] | Congo, 2011-2012 | 477 | National | Hb, <11 g/dl | 58.4 | 279 |
|  | Institut National de la Statistique, 2013 [38] | Guinea, 2013 | 492 | National | Hb, <11 g/dl | 64.9 | 319 |
|  | INS, 2013 [48] | Niger, 2012 | 743 | National | Hb, <11 g/dl | 58.6 | 435 |
| AMR | MSPAS, 2017 [16] | Guatemala, 2014-2015 | 1408 | National | Hb, <11 g/dl | 24.2 | 341 |
|  | Cayemittes, 2013 [41] | Haiti, 2012 | 524 | National | Hb, <11 g/dl | 53.9 | 282 |
|  | Honduras Secretaria de Salud, 2013 [42] | Honduras, 2012 | 1146 | National | Hb, <11 g/dl | 18.8 | 215 |
| SEAR | Ministry of Health and Sports, 2017 [43] | Myanmar, 2015-2016 | 449 | National | Hb, <11 g/dl | 56.9 | 256 |
|  | Bhutan Ministry of Health, 2015 [44] | Bhutan, 2015 | 148 | National | Hb, <11 g/dl | 27.3 | 41 |
|  | Ministry of Health, 2013 [49] | Indonesia, 2013 | 503 | National | Hb, <11 g/dl | 37.1 | 187 |
|  | Nepal Ministry of Health and Population, 2013 [45] | Nepal, 2012 | 293 | National | Hb, <11 g/dl | 47.6 | 140 |
| EMR | Ministry of Health and Population, 2014 [46] | Egypt, 2014 | 727 | National | Hb, <11 g/dl | 20.6 | 150 |
| WPR | National Institute of Statistics, 2015 [14] | Cambodia, 2014 | 615 | National | Hb, <11 g/dl | 53.2 | 327 |
|  | Food and Nutrition Research Institute, (2015) [50] | Philippines, 2013 | 1196 | National | Hb, <11 g/dl | 24.6 | 294 |

AFR: Africa; AMR: America; SEAR: South East Asia; EUR: Europe; EMR: Eastern Mediterranean; WPR: Western Pacific

*References*

1. Ethiopian Public Health Institute, *Ethiopian National Micronutrient Survey.* 2016.

2. Alaofe, H., et al., *Prevalence of anaemia, deficiencies of iron and vitamin A and their determinants in rural women and young children: a cross-sectional study in Kalale district of northern Benin.* Public Health Nutr, 2017. **20**(7): p. 1203-1213.

3. Liberia Institute of Statistics and UNICEF, *Liberia Natonal Nutrition Survey 2011.* 2011.

4. Cameroon Ministry of Public Health, et al., *National Survey of Micronutrient and Habit of Fortifable Food Consumption*. 2009.

5. Wirth, J.P., et al., *Predictors of anemia in women of reproductive age: Biomarkers Reflecting Inflammation and Nutritional Determinants of Anemia (BRINDA) project.* Am J Clin Nutr, 2017. **106**(Suppl 1): p. 416s-427s.

6. International Institute of Tropical Agriculture (IITA), et al., *Nigeria Food Consumption and Nutrition Survey 2001-2003.* 2004.

7. Timor-Leste Ministry of Health, *Timor-Leste Food and Nutrition Survey in 2013*. 2015.

8. International Centre for Diarrheal Diseases Research, et al., *National Micronutrient Status Survey Bangladesh 2011-2012.* 2013.

9. Madanijah, S., et al., *Nutritional status of lactating women in Bogor district, Indonesia: cross-sectional dietary intake in three economic quintiles and comparison with pre-pregnant women.* Br J Nutr, 2016. **116 Suppl 1**: p. S67-74.

10. Kyrgyzstan Ministry of Health, et al., *National Survey of the Nutritional Status of Children 6-59 Months of Age and Their Mothers.* 2009.

11. Ministry of Public Health and UNICEF Afghanistan, *National Nutrition Survey Afghanistan.* 2013.

12. Aga Khan University, *Pakistan National Nutrition Survey.* 2011.

13. Ministry of Health, et al., *National Micronutrient Survey Jordan 2010.* 2011.

14. National Institute of Statistics, Directorate General for Health, and I. International, *Cambodia Demographic and Health Survey 2014*. 2015: Phnom Penh, Cambodia, and Rockville, Maryland, USA: National Institute of Statistics, Directorate General for Health, and ICF International.

15. Laillou, A., et al., *Micronutrient deficits are still public health issues among women and young children in Vietnam.* PLoS One, 2012. **7**(4): p. e34906.

16. MSPAS, et al., *Encuesta Nacional de Micronutrientes 2009-2010.* 2012.

17. UNICEF, *Georgia National Nutrition Survey 2009.* 2010.

18. Clewes, C., et al., *LC‐LQAS SURVEY REPORT.* 2012.

19. Tawfik, A., E. Hanna, and S. Freig, *Folate Status in Egypt.* IOSR J. Nurs. Health Sci, 2014. **3**(2): p. 32–36.

20. Bromage, S., T. Ahmed, and W.W. Fawzi, *Calcium Deficiency in Bangladesh: Burden and Proposed Solutions for the First 1000 Days.* Food Nutr Bull, 2016. **37**(4): p. 475-493.

21. Uush, T., *Prevalence of classic signs and symptoms of rickets and vitamin D deficiency in Mongolian children and women.* J Steroid Biochem Mol Biol, 2013. **136**: p. 207-10.

22. Smith, G., et al., *High Prevalence of Vitamin D Deficiency in Cambodian Women: A Common Deficiency in a Sunny Country.* Nutrients, 2016. **8**(5).

23. UNICEF, Irish Aid, and CDC, *Malawi National Micronutrient Survey.* 2009.

24. FSNAU, FAO, and UCL, *National Micronutrient and Anthropometric Nutrition Survey Somalia.* 2009.

25. Tajikistan Ministry of Health and UNICEF, *Micronutrient Status Survey in Tajikistan, 2009.* 2009.

26. Hess, S.Y., *National Risk of Zinc Deficiency as Estimated by National Surveys.* Food Nutr Bull, 2017: p. 379572116689000.

27. Engle-Stone, R., et al., *Stunting prevalence, plasma zinc concentrations, and dietary zinc intakes in a nationally representative sample suggest a high risk of zinc deficiency among women and young children in Cameroon.* J Nutr, 2014. **144**(3): p. 382-91.

28. Wieringa, F.T., et al., *The High Prevalence of Anemia in Cambodian Children and Women Cannot Be Satisfactorily Explained by Nutritional Deficiencies or Hemoglobin Disorders.* Nutrients, 2016. **8**(6).

29. Marcos, J.M., et al., *Serum Zinc Levels in Selected Filipino Population Groups.* 2008.

30. Petry, N., et al., *The Proportion of Anemia Associated with Iron Deficiency in Low, Medium, and High Human Development Index Countries: A Systematic Analysis of National Surveys.* Nutrients, 2016. **8**(11).

31. Rwanda National Institute of Statistics of Rwanda (NISR), Ministry of Health (MOH) [Rwanda], and I. International, *Rwanda Demographic and Health Survey 2014-15*. 2015, NISR, MOH, and ICF International: Rockville, Maryland, USA.

32. Lesotho Ministry of Health and I. International, *Lesotho Demographic and Health Survey 2014*. 2016, Ministry of Health and ICF International: Maseru, Lesotho.

33. Ghana Statistical Service (GSS), Ghana Health Service (GHS), and ICF International, *Ghana Demographic and Health Survey 2014.* 2015.

34. Ministère de la Planification, et al., *[Togo National Demographic Health Survey 2013-2014]*. 2015, MPDAT, MS et ICF International: Rockville, Maryland, USA.

35. National Institute of Statistics, *Sao Tome and Principe Multiple Indicator Cluster Survey 2014, Final Report*. 2016: São Tomé, Sao Tome and Principe.

36. The Nambia Ministry of Health and Social Services (MoHSS) and ICF International, *The Namibia Demographic and Health Survey 2013*. 2014, MoHSS and ICF: Windhoek, Namibia, and Rockville, Maryland, USA.

37. Ministère du Plan et Suivi de la Mise en œuvre de la Révolution de la Modernité (MPSMRM), Ministère de la Santé Publique (MSP), and I. International, *[Democratic Republic of Congo National Demographic and Health Survey 2012-2013]*. 2014, MPSMRM, MSP et ICF International: Rockville, Maryland, USA.

38. Institut National de la Statistique, et al., *[Guinea Demographic and Health Survey and Multiple Indicators 2012]*. 2013: Conakry, Guinée and Maryland, U.S.A.

39. Institut National de la Statistique et de l’Analyse Économique (INSAE) and I. International, *[Benin National Demographic and Health Survey 2011-2012]*. 2013, INSAE et ICF International: Calverton, Maryland, USA.

40. Congo Centre Nationale de la Statistique et des Études Économiques (CNSEE) and I. International, *[Congo National Demographic and Health Survey 2011-2012]*. 2013, CNSEE and ICF International: Calverton, Maryland, USA.

41. Cayemittes, et al., *[Mortality, Morbidity and Service Usage Survey in Haiti 2012]*. 2013, MSPP, IHE and ICF International: Calverton, Maryland, USA.

42. Honduras Secretaría de Salud, Instituto Nacional de Estadística (INE), and M. International, *[Honduras National Demographic and Health Survey 2011-2012]*. 2013, SS, INE e ICF International: Tegucigalpa, Honduras.

43. Ministry of Health and Sports and ICF, *Myanmar Demographic and Health Survey 2015-16*. 2017, Ministry of Health and Sports and ICF: Nay Pyi Taw, Myanmar, and Rockville, Maryland USA.

44. Bhutan Ministry of Health, *Bhutan National Nutrition Survey*. 2015, Ministry of Health: Thimphu, Bhutan.

45. Nepal Ministry of Health and Population (MOHP), New ERA, and I. International, *Nepal Demographic and Health Survey 2011*. 2012, Ministry of Health and Population, New ERA, and

ICF International, Calverton, Maryland.: Kathmandu, Nepal.

46. Egypt Ministry of Health and Population, *Egypt Demographic and Health Survey 2014*. 2014, Ministry of Health and Population and ICF International: Cairo, Egypt and Rockville, Maryland, USA.

47. Jordan Department of Statistics, et al., *Jordan Population and Family Health Survey 2012*. 2013, Department of Statistics and ICF International: Calverton, Maryland, USA.

48. Institut National de la Statistique (INS) and I. International, *[Niger National Demographic and Health Survey and Multiple Indicator, 2012]*. 2013, INS et ICF International: Calverton, Maryland, USA.

49. Ministry of Health, *[Indonesia Basic Health Survey].* 2013.

50. Food and Nutrition Research Institute, *Philippine Nutrition Facts and Figures 2013: 8th National Nutrition Survey Overview.* 2015.
