# Supplementary material for: Review of the evidence regarding the use of antenatal multiple micronutrient supplementation in low‐ and middle‐income countries
Source: Ann N Y Acad Sci. 2019 May 27;1444(1):6–21. doi: 10.1111/nyas.14121 (PMC6852202; doi:10.1111/nyas.14121)
Supplement: Supplementary file 4 — Table S4. Vitamin deficiencies among pregnant women in low‐ and middle‐income countries (LMICs) Table S5. Mineral deficiencies among pregnant women in low‐ and middle‐income countries (LMICs) [file NYAS-1444-6-s004.docx]

**Prevalence of micronutrient deficiencies in pregnancy**

**Table S4. Vitamin deficiencies among pregnant women in Low- and Middle-Income Countries (LMIC)**

| **Region** | **First author, publication year** | **Country, year of field study** | **N** | **Setting of study population** | **Biomarker, vitamin cut-off** | **Vitamin deficiency, %** | **Vitamin deficiency (n)** |
| --- | --- | --- | --- | --- | --- | --- | --- |
| **Vitamin A** |  |  |  |  |  |  |  |
| AFR | International Institute of Tropical Agriculture, 2004 [1] | Nigeria, 2001-2003 | 684 | National | Serum retinol <20 µg/dl | 8.8 | 60 |
| SEAR | Madanijah, 2016^a^ [2] | Indonesia, 2010-2011 | 45 | Local, Bogor District | Serum retinol <200 µg/l | 11.1 | 5 |
|  | Jiang, 2005 [3] | Nepal, 1998-2001 | 1165 | National | Serum retinol, <0·70 µmol/l | 6.8 | 79 |
| EMR | Aga Khan University, 2011 [4] | Pakistan, 2011 | 912 | National | Serum retinol, <0.70 µmol/l | 48.7 | 444 |
|  | Hwalla, 2016 [5] | Egypt, 1995-2005 | 1845 | National | Serum retinol, <0.70 µmol/l | 21.5 | 397 |
|  | Hwalla, 2016 [5] | Jordan, 1995-2005 | 155 | National | Serum retinol, <0.70 µmol/l | 24.2 | 38 |
| **Vitamin B-2 (Riboflavin)** |  |  |  |  |  |  |  |
| SEAR | Jiang, 2005 [3] | Nepal, 1998-2001 | 1163 | National | Riboflavin, <11.3 nmol/l | 31.8 | 370 |
| **Vitamin B-6 (Pyridoxine)** |  |  |  |  |  |  |  |
| SEAR | Jiang, 2005 [3] | Nepal, 1998-2001 | 1164 | National | Pyridoxal-5’-phosphate, <19 nmol/l | 40.3 | 469 |
| **Vitamin B-9 (Folate)** |  |  |  |  |  |  |  |
| SEAR | Gernand, 2016 [6] | South Asia: India, Bangladesh, Nepal, 2016 | N/R | Regional | Serum, < 6.7 nmol/l | 0-26 | N/R |
|  | Jiang, 2005 [3] | Nepal, 1998-2001 | 1164 | National | Serum, < 6.7 nmol/l | 11.1 | 134 |
| **Vitamin B-12 (Cobalamine)** |  |  |  |  |  |  |  |
| SEAR | Gernand, 2016 [6] | South Asia : India, Bangladesh, Nepal, N/R | N/R | Regional | Serum, <150 pmol/l | 19-74 | N/R |
|  | Jiang, 2005 [3] | Nepal, 1998-2001 | 1158 | National | Serum, <150 pmol/l | 28.3 | 328 |
| **Vitamin D** |  |  |  |  |  |  |  |
| AFR | Luxwolda, 2013 in Palacios, 2014 [7] | Tanzania, 2003-2009 | 139 | Local, Dar-Es-Salam | Serum, <50 nmol/l | 1 | 1 |
| SEAR | Jiang, 2005 [3] | Nepal, 1998-2001 | 1163 | National | Serum, <25nmol/l | 13.9 | 162 |
|  | Trilok Kumar, 2015 [8] | India, 2000-2015 | 2235 | National | Serum, <50nmol/l | 67 | 1498 |
| EMR | El Rifai, 2014 [9] | Egypt, 2012-2013 | 135 | Local, Cairo | Serum, ≤20 ng/ml | 40 | 54 |
|  | Aga Khan University, 2011 [4] | Pakistan, 2011 | 699 | National | Serum, ≤20 ng/ml | 68.5 | 479 |
| **Vitamin E** |  |  |  |  |  |  |  |
| AFR | International Institute of Tropical Agriculture, 2004 [1] | Nigeria, 2001-2003 | 684 | National | Tocopherol, <5 µg/dl | 12 | 83 |
| SEAR | Gernand, 2016 [6] | South Asia: India, Bangladesh, Nepal, N/R | N/R | Regional | α-tocopherol <12.0 μmol/l | 50-70 | N/R |
|  | Jiang, 2005 [3] | Nepal, 1998-2001 | 1165 | National | α-tocopherol <9.3 µmol/l | 25.3 | 293 |

N/R: Not reported; AFR: Africa; AMR: America; SEAR: South East Asia; EUR: Europe; EMR: Eastern Mediterranean; WPR: Western Pacific

1. Lactating women

**Table S5. Mineral deficiencies among pregnant women in Low- and Middle-Income Countries (LMIC)**

| **Region** | **First author, publication year** | **Country, year of field study** | **N** | **Setting of study population** | **Biomarker, mineral cut-off** | **Mineral deficiency, %** | **Mineral deficiency (n)** |
| --- | --- | --- | --- | --- | --- | --- | --- |
| **Zinc** |  |  |  |  |  |  |  |
| AFR | International Institute of Tropical Agriculture, 2004 [1] | Nigeria, 2001-2003 | 795 | National | Plasma, <80µg/dl | 43.8 | 348 |
| SEAR | Madanijah, 2016^a^ [2] | Indonesia, 2010-2011 | 45 | Local, Bogor District | Plasma, <65 µmol/l | 24.4 | 11 |
|  | Pathak, 2008 [10] | India, 2000-2001 | 257 | Local, a rural block, Haryana state | Serum, <66 µg/dl | 64.6 | 166 |
|  | Jiang, 2005 [3] | Nepal, 1998-2001 | 1165 | National | Zinc, <8.6 µmol/l | 61.1 | 712 |
|  | Gernand, 2016 [6] | South Asia: India, Bangladesh, Nepal, N/R | N/R | Regional | Serum, <66 µmol/l | 15-74 | N/R |
| EMR | Aga Khan University, 2011[4] | Pakistan, 2011 | 791 | National | Plasma, <60µg/dl | 48.3 | 382 |
| WPR | Marcos, 2008^a^ [11] | Philippines, 2008 | 836 | National | IZiNCG cutoffs^a^ | 39.7 | 332 |
| **Iron Deficiency** | | | | | | | |
| AFR | International Institute of Tropical Agriculture, 2004 [1] | Nigeria, 2001-2003 | 829 | National | Serum ferritin, <12ng/ml | 19.9 | 165 |
| EMR | Aga Khan University, 2011[4] | Pakistan, 2011 | 982 | National | Serum Ferritin, <12 ng/dl | 38.2 | 375 |
| SEAR | Madanijah, 2016^a^ [2] | Indonesia, 2010-2011 | 45 | Local, Bogor District | Serum ferritin, ≤15 µg/l | 27.9 | 13 |
|  | Jiang, 2005 [3] | Nepal, 1998-2001 | 1163 | National | Serum ferritin <10 µg/l | 39.8 | 463 |
| **Iron Deficiency Anemia (IDA)^b^** | | | | | | | |
| AFR | Black, 2013 [12] | Africa, 2011 | N/R | Regional | Hb, <110 g/l | 20.3 | N/R |
| AMR | Black, 2013 [12] | Americas and the Caribbean, 2011 | N/R | Regional | Hb, <110 g/l | 15.2 | N/R |
| SEAR | Black, 2013 [12] | Asia, 2011 | N/R | Regional | Hb, <110 g/l | 19.8 | N/R |
| EUR | Black, 2013 [12] | Europe, 2011 | N/R | Regional | Hb, <110 g/l | 16.2 | N/R |
| WPR | Black, 2013 [12] | Oceania, 2011 | N/R | Regional | Hb, <110 g/l | 17.2 | N/R |
| WORLD | Black, 2013 [12] | Worldwide, 2011 | N/R | Global | Hb, <110 g/l | 19.2 | N/R |
| **Selenium** |  |  |  |  |  |  |  |
| SEAR | Madanijah, 2016^a^ [2] | Indonesia, 2010-2011 | 45 | Local, Bogor District | Plasma, <0.75µmol/l | 28.9 | 13 |
| **Calcium** |  |  |  |  |  |  |  |
| EMR | Aga Khan University, 2011 [4] | Pakistan, 2011 | 1048 | National | <8.4 mg/dl | 58.3 | 611 |

N/R: Not reported; AFR: Africa; AMR: America; SEAR: South East Asia; EUR: Europe; EMR: Eastern Mediterranean; WPR: Western Pacific

1. Lactating women
2. Estimated from 50% of anaemia in pregnant women caused by IDA

*References*

1. International Institute of Tropical Agriculture (IITA), et al., *Nigeria Food Consumption and Nutrition Survey 2001-2003.* 2004.

2. Madanijah, S., et al., *Nutritional status of lactating women in Bogor district, Indonesia: cross-sectional dietary intake in three economic quintiles and comparison with pre-pregnant women.* Br J Nutr, 2016. **116 Suppl 1**: p. S67-74.

3. Jiang, T., et al., *Micronutrient deficiencies in early pregnancy are common, concurrent, and vary by season among rural Nepali pregnant women.* J Nutr, 2005. **135**(5): p. 1106-12.

4. Aga Khan University, *Pakistan National Nutrition Survey.* 2011.

5. Hwalla, N., et al., *The Prevalence of Micronutrient Deficiencies and Inadequacies in the Middle East and Approaches to Interventions.* Nutrients, 2017. **9**(3).

6. Gernand, A.D., et al., *Micronutrient deficiencies in pregnancy worldwide: health effects and prevention.* Nat Rev Endocrinol, 2016. **12**(5): p. 274-89.

7. Palacios, C. and L. Gonzalez, *Is vitamin D deficiency a major global public health problem?* J Steroid Biochem Mol Biol, 2014. **144 Pt A**: p. 138-45.

8. Trilok Kumar, G., R. Chugh, and M. Eggersdorfer, *Poor Vitamin D Status in Healthy Populations in India: A Review of Current Evidence.* Int J Vitam Nutr Res, 2015. **85**(3-4): p. 185-201.

9. El Rifai, N.M., et al., *Vitamin D deficiency in Egyptian mothers and their neonates and possible related factors.* J Matern Fetal Neonatal Med, 2014. **27**(10): p. 1064-8.

10. Pathak, P., et al., *Serum zinc levels amongst pregnant women in a rural block of Haryana state, India.* Asia Pac J Clin Nutr, 2008. **17**(2): p. 276-9.

11. Marcos, J.M., et al., *Serum Zinc Levels in Selected Filipino Population Groups.* 2008.

12. Black, R.E., et al., *Maternal and child undernutrition and overweight in low-income and middle-income countries.* Lancet, 2013. **382**(9890): p. 427-51.
